# Endometriotic cyst mimicking recurrence after treatment for ovarian immature teratoma: a case report

**DOI:** 10.3389/fonc.2026.1846023

**Published:** 2026-06-09

**Authors:** Ying Dong, Yue Deng, Youfang Hou, Rongji Li, Lihua Yang

**Affiliations:** Department of Gynaecology, The Second Affiliated Hospital of Kunming Medical University, Kunming, Yunnan, China

**Keywords:** case report, endometriosis, GnRH-a, immature teratoma, ovarian endometriotic cyst

## Abstract

**Background:**

Endometriosis is common in reproductive-age women, but the development of an endometriotic cyst that mimics tumor recurrence during follow-up after treatment for ovarian immature teratoma poses a significant diagnostic challenge, particularly for young patients requiring fertility preservation.

**Case presentation:**

A 23-year-old woman with a history of two fertility-sparing surgeries and BEP (bleomycin, etoposide, cisplatin) chemotherapy for ovarian immature teratoma was followed up. Pelvic MRI revealed a 5.7×4.5cm left adnexal multicystic lesion, suggestive of hemorrhagic content, and without restricted diffusion. Tumor markers (CA125, CA199, AFP, CEA) remained within normal limits. Although a clinical diagnosis of endometriotic cyst was considered, the possibility of recurrence could not be completely ruled out. Given the high risk of irreversible ovarian damage from a third surgery and the patient’s refusal of needle biopsy, a trial of gonadotropin-releasing hormone agonist (GnRH-a) therapy was initiated. After three cycles, the mass markedly decreased to 1.4 × 0.9 cm. Maintenance dienogest was subsequently given. At one-year follow-up, there was no evidence of disease progression; the patient maintained regular menstrual cycles and ovarian reserve was preserved (anti-Müllerian hormone (AMH) >2ng/mL), supporting a clinical diagnosis consistent with an endometriotic cyst.

**Conclusion:**

This case suggests that, in carefully selected patients with reassuring clinical, imaging, and laboratory findings and under close oncologic surveillance, a therapeutic trial of hormonal therapy may serve as an alternative to immediate surgical exploration for a newly developed adnexal mass after treatment for malignant ovarian tumors, especially when fertility preservation is a priority.

## Introduction

Among benign gynecological conditions affecting reproductive-age women, endometriosis ranks as one of the most prevalent. It is a chronic, estrogen-dependent condition. The prevalence of endometriosis in the general population is approximately 2–10%, but in infertile women of reproductive age, it can be as high as 35% (1). Endometriosis shares certain biological features with malignant processes, including progressive growth and a tendency for local recurrence, which can complicate clinical surveillance (2). Ovarian malignant germ cell tumors constitute a rare and heterogeneous group, accounting for approximately 5% of all ovarian malignancies. They predominantly affect women under 30 years of age, and a considerable proportion of recurrences occur within the first two years after initial diagnosis (3, 4). For patients with a history of ovarian germ cell tumors, the detection of a new adnexal mass during follow-up raises a critical differential diagnosis between tumor recurrence and a benign lesion such as an endometriotic cyst. This diagnostic challenge is particularly relevant in young women for whom repeated surgery may compromise ovarian reserve and fertility potential.

This report describes a 23-year-old woman who underwent two fertility-sparing surgeries and BEP chemotherapy for high-grade immature teratoma of the ovary. During follow-up, a newly developed adnexal mass was detected. Based on comprehensive clinical, laboratory, and imaging assessment, a therapeutic trial of a gonadotropin-releasing hormone agonist (GnRH-a) was employed to distinguish, on clinical grounds, an endometriotic cyst from tumor recurrence, while aiming to preserve ovarian function as evidenced by regular menstrual cycles and anti-Müllerian hormone (AMH) levels within the normal range. Given the absence of histological confirmation, the diagnosis remains clinical.

Clinical experience regarding this specific scenario is extremely limited, particularly with respect to conservative management strategies that balance diagnostic accuracy with fertility preservation in patients with a history of ovarian malignancy. This case aims to explore the differential diagnostic approach for newly developed adnexal masses detected during follow-up after treatment for malignant ovarian germ cell tumors, and to discuss an individualized management strategy that prioritizes fertility preservation under close surveillance.

## Case presentation

The reporting of this study complies with the CARE guidelines, and written informed consent was obtained from the patient.

A 23-year-old female patient presented in November 2020 with persistent right lower abdominal pain. Imaging revealed a large pelvic mass (approximately 22×20×18cm³) with elevated tumor markers (CA125, CA199, AFP, CEA above normal limits). She was admitted to the Department of Gynecology at an affiliated hospital of a medical university in China. Following evaluation, she underwent cytoreductive surgery for a large right pelvic mass(R0), resection of the right adnexa, lesions in the rectouterine pouch and vesicouterine pouch, omentum, and pelvic adhesiolysis. Postoperative pathology confirmed a high-grade immature teratoma of the right ovary, FIGO stage IIB. However, The patient declined adjuvant chemotherapy and regular follow-up.

In June 2021, the patient returned with lower abdominal pain. Physical examination noted a 7 cm tender mass, and CT and pelvic ultrasound revealed a 6.8 × 5.8cm cystic-solid mass in the right pelvic region. Tumor markers showed AFP<2 ng/mL, CEA 0.73 ng/mL, CA199 18.43 U/mL (all normal), and CA125 226.3 U/mL (elevated). Transrectal ultrasonography confirmed a 5.4×4.7cm cystic mass with septations and blood flow signals. A clinical diagnosis of recurrent ovarian immature teratoma was made, and two cycles of neoadjuvant BEP (bleomycin, etoposide, cisplatin) chemotherapy were administered. In August 2021, laparotomy revealed numerous white miliary nodules on the bowel surface, a 7×6×5cm irregular cystic-solid tumor of the left ovary, and a small amount of gelatinous pelvic fluid. Left ovarian tumor excision was performed with preservation of normal ovarian tissue. Postoperative pathology confirmed a high-grade immature teratoma of the left ovary. Two additional cycles of BEP chemotherapy were given, with a GnRH-a administered every 28 days for ovarian protection during treatment. Thereafter, the patient was followed regularly. Menstrual cycles returned to normal, with occasional tolerable dysmenorrhea, and serial tumor markers (CA125, CA199, AFP, CEA) remained within normal limits.

**Figure 1:**
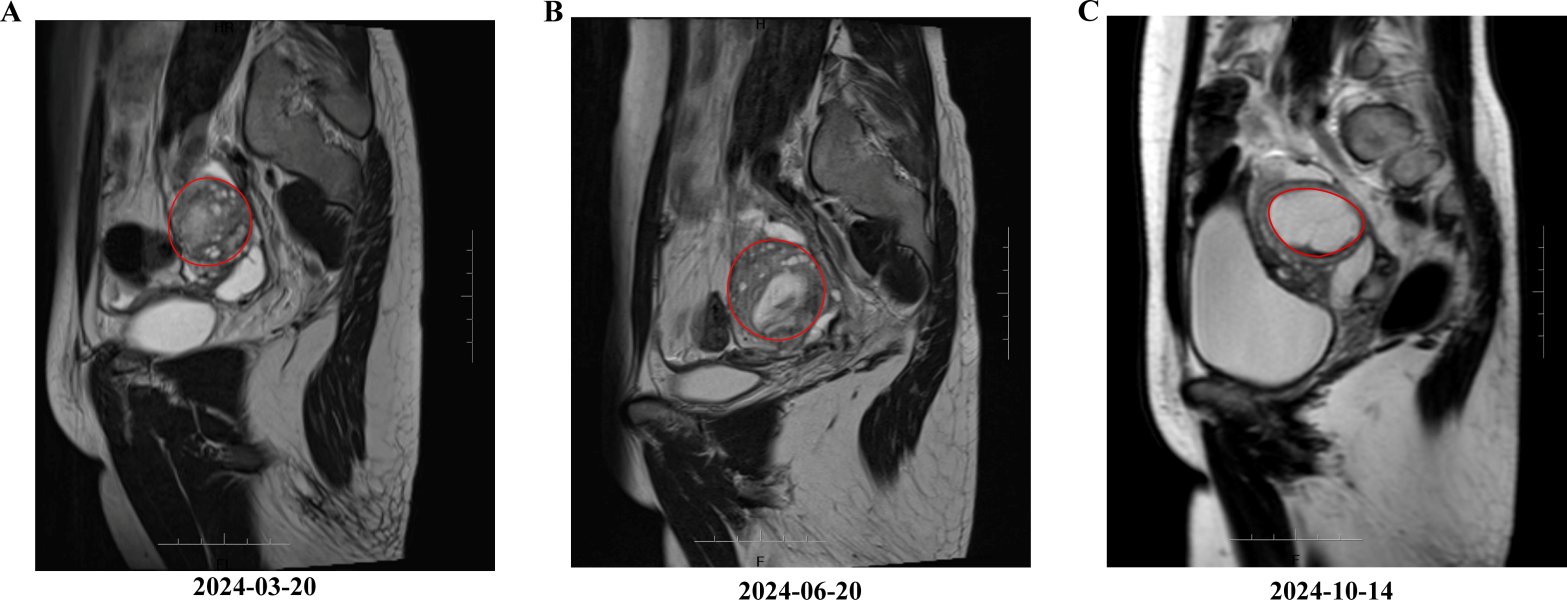
Pelvic MRI of the left ovarian mass from March to October 2024. **(A)** March 2024: Sagittal T2-weighted fast spin-echo sequence (TE 83 ms, TR 5390 ms, slice thickness 4.0 mm) showing a 5.7×4.5×3.8 cm multicystic lesion in the left adnexal region (red circle) with internal septations and features suggestive of hemorrhage. **(B)** June 2024: Sagittal T2-weighted fast spin-echo sequence (TE 83 ms, TR 4600 ms, slice thickness 4.5 mm) showing a 3.7×3.7×4.7 cm multicystic lesion in the left adnexal region (red circle) with internal septations and signal characteristics suggestive of hemorrhage. **(C)** October 2024: Sagittal breath-hold T2-weighted turbo spin-echo sequence (TE 80 ms, TR 850 ms, slice thickness 4.5 mm) showing a 5.7×4.5×3.8cm cystic lesion in the left adnexal region (red circle), suggestive of hemorrhage.

**Figure 2:**
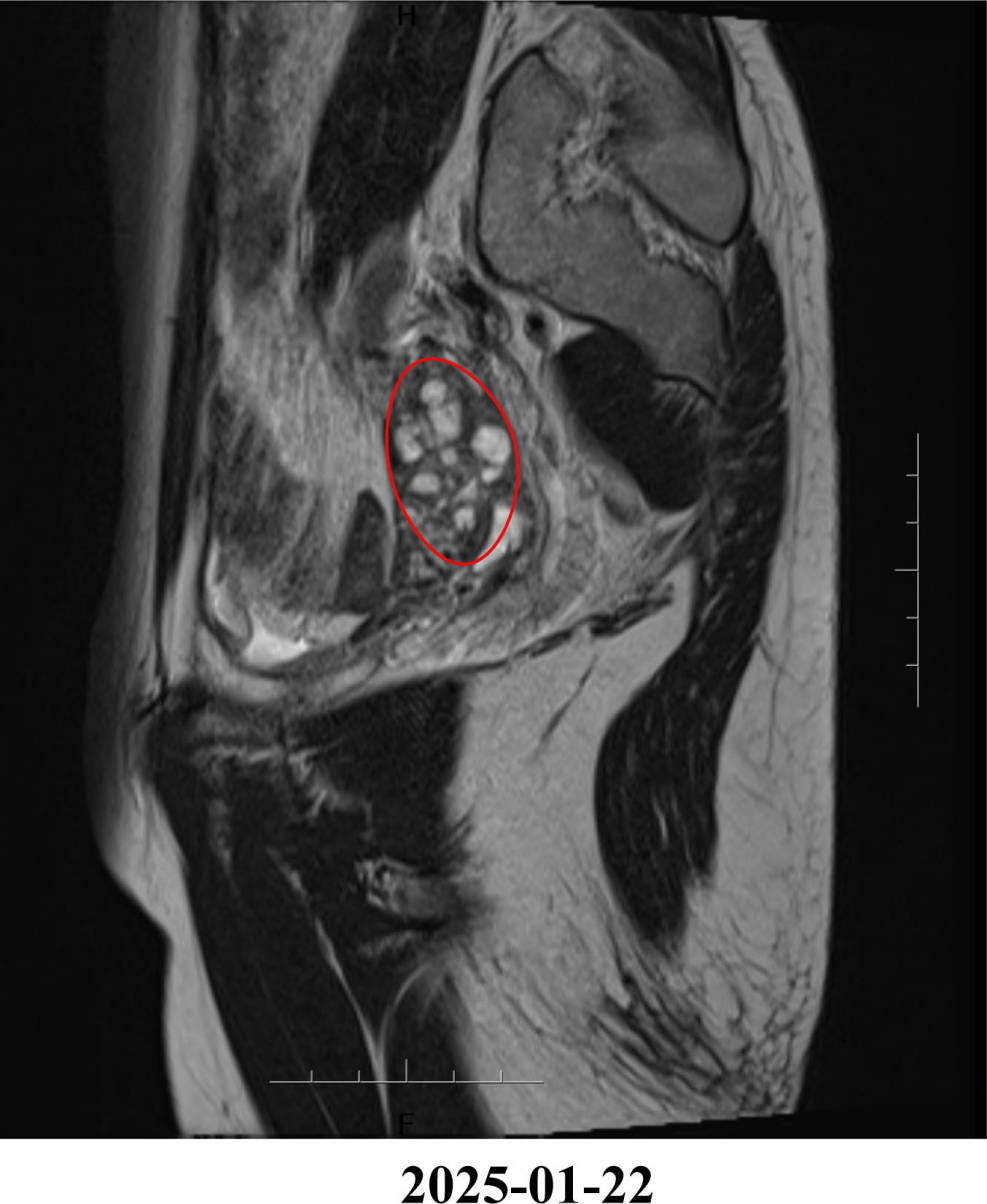
Pelvic MRI in January 2025. Sagittal T2-weighted fast spin-echo sequence (TE 83 ms, TR 5990 ms, slice thickness 4.0 mm) showing multiple small cystic lesions in the left adnexal region.

In March 2024, routine MRI revealed an enlarged left ovary with focal hemorrhagic signal, suspicious for a chocolate cyst but not excluding recurrence. The follow-up interval was shortened to every three months. In March 2024, MRI showed a 5.7×4.5×3.8 cm multicystic lesion in the left adnexa (**Figure 1A**). In June 2024, repeat MRI demonstrated a reduction in size to 3.7×3.7×4.7 cm (**Figure 1B**). By October 2024, the mass had re-enlarged to 5.7×4.5×3.8 cm (**Figure 1C**). The lesion appeared as a multicystic mass with internal septations, predominantly long T1/long T2 signal, and focal short T1 signal suggestive of hemorrhage; these features were consistent with an endometriotic cyst. Tumor markers showed AFP<1.8ng/mL, CEA<0.5ng/mL, CA199 10.09U/mL, and CA125 11.96 U/mL, all remained within normal limits. Given the patient’s two prior ovarian surgeries, the high risk of ovarian function loss from a third surgery, and her firm refusal of needle biopsy, a multidisciplinary discussion concluded the mass was highly suggestive of an endometriotic cyst, and a therapeutic trial of a GnRH-a was initiated (last dose December 2024). In January 2025, MRI showed near-complete resolution of the left adnexal mass (**Figure 2**), leaving only small long T1/long T2 cysts (largest 0.7 cm) and minimal residual fluid, interpreted as physiological cysts and residual hemorrhagic/serous fluid. Given persistently normal tumor markers and preserved ovarian function, this regression was consistent with a benign process such as a resolving endometriotic cyst rather than tumor recurrence. Dienogest (2 mg/day) was started as maintenance therapy. At the last follow-up (January 2026), there was no evidence of disease progression, the patient maintained regular menstrual cycles, tumor markers remained normal, and ovarian function was preserved (AMH 2.5 ng/mL). 

## Discussion

Endometriosis is one of the most common benign gynecological conditions in women of reproductive age, and ovarian endometrioma accounts for 17-44% of benign ovarian cysts (5). Accumulating evidence also suggests an association between endometriosis and ovarian cancer; for instance, Siufi et al. found that 1.1% of women with endometriosis developed histologically confirmed ovarian cancer (6). In the present case, however, the clinical question was reversed: a patient with a history of prior ovarian immature teratoma surgeries and chemotherapy developed a gradually enlarging adnexal mass during follow-up, raising concern for tumor recurrence. The key diagnostic dilemma was distinguishing recurrence from a newly developed benign endometriotic cyst. Reports describing endometriotic cysts that mimic recurrence in an oncologic follow-up setting are exceedingly rare, and the optimal management strategy for young women requiring fertility preservation remains undefined.

Currently, the diagnosis of endometriosis is often clinical, and definitive diagnosis is challenging, frequently leading to delays (7, 8). Surgery remains the gold standard for confirmation, but it requires weighing the risk of surgical complications against the potential decrease in ovarian reserve (9). In this patient, who had a clear history of recurrent ovarian immature teratoma, tumor recurrence was undoubtedly the most critical consideration. However, she had no tumor-related symptoms during follow-up, key tumor markers remained persistently normal, and pelvic MRI findings were highly suggestive of an endometriotic cyst, making a benign lesion a strong possibility. The decision for surgical exploration therefore had to be balanced against the fact that she had already undergone two ovarian surgeries and had no history of childbirth, such that a third procedure carried a risk of irreversible ovarian function decline. Although the posterior location of the mass made transvaginal ultrasound-guided needle biopsy a potential diagnostic option, the patient firmly refused the procedure. Given the impracticality of invasive tissue sampling, imaging became the central diagnostic tool. Among available modalities, MRI offers relatively high diagnostic accuracy for endometriosis, with a reported sensitivity of 82% and specificity of 87% (10). Following multidisciplinary discussion with the radiology department, the imaging characteristics, together with the patient’s history of dysmenorrhea, were considered most consistent with an endometriotic cyst. It should be acknowledged, however, that without histological confirmation, the diagnosis remains clinical, and even reassuring tumor markers and imaging findings cannot completely rule out recurrence. This residual uncertainty, together with the imperative to preserve fertility, made a therapeutic trial a next step.

In light of this uncertainty, a GnRH-a trial was considered a reasonable next step. Although first-line hormonal therapy for endometriosis typically includes contraceptives and nonsteroidal anti-inflammatory drugs (11), GnRH-as are more often reserved for second-line use (12). The 2019 NCCN guidelines additionally note their ovarian-protective potential during chemotherapy, a consideration relevant to this patient who had previously received gonadotoxic agents. In the present case, the suspicion of an endometriotic cyst, the imperative to preserve fertility, and the risks of reoperation collectively made a trial of GnRH-a therapy preferable to immediate surgical exploration. After three cycles, follow-up imaging demonstrated a marked reduction of the mass, a response that strongly supported an endometriotic cyst rather than a chemotherapy-insensitive recurrence. The patient subsequently received maintenance dienogest and, at one-year follow-up, had no evidence of disease progression, with regular menstrual cycles and preserved ovarian function, further supporting the clinical diagnosis.

This case has several limitations. First, the absence of histopathological confirmation is the most important, as the diagnosis of an endometriotic cyst remains clinical. Second, this is a single case report, and the generalizability of this conservative approach is limited. Third, although the one-year follow-up has shown no evidence of disease progression, longer observation is needed to confirm long-term outcomes.

Overall, this case suggests that a conservative approach with hormonal therapy may be considered as an alternative to immediate surgical exploration for a newly detected adnexal mass during follow-up after treatment for malignant ovarian germ cell tumors, provided that patients are carefully selected on the basis of reassuring imaging features, persistently normal tumor markers, and multidisciplinary consensus. However, this strategy requires individualized assessment and close oncologic surveillance and cannot be generalized based on a single case.

## Conclusion

This case describes a reproductive-age woman with a history of prior ovarian immature teratoma surgeries and chemotherapy who developed a newly detected adnexal mass during follow-up, raising the differential diagnosis between tumor recurrence and a benign endometriotic cyst. Given the impracticality of invasive tissue sampling and the paramount need for fertility preservation, a multidisciplinary approach integrating clinical findings, persistently normal tumor markers, and MRI features was adopted, and a therapeutic trial of a GnRH-a was initiated. The marked radiological regression supported a clinical diagnosis consistent with an endometriotic cyst rather than recurrence. However, the absence of histopathological confirmation represents a key limitation, and the diagnosis remains clinical. This case suggests that, in carefully selected patients with reassuring imaging and laboratory findings and under close oncologic surveillance, a conservative approach with hormonal therapy may be considered as an alternative to immediate surgical exploration. This strategy requires individualized multidisciplinary assessment and cannot be generalized based on a single case report. Longer follow-up is needed to confirm long-term outcomes.

## Data Availability

The original contributions presented in the study are included in the article/supplementary material. Further inquiries can be directed to the corresponding author.
